# Gallbladder adenocarcinoma skin metastasis

**DOI:** 10.4322/acr.2023.458

**Published:** 2023-11-27

**Authors:** Arvind Kumar, Tushar Kalonia, Akanksha Gupta, Ravi Hari Phulware

**Affiliations:** 1 All India Institute of Medical Sciences (AIIMS), Department of Pathology, Laboratory Medicine, Rishikesh, Uttarakhand, India

**Keywords:** Gallbladder Neoplasm, Skin Ulcer, Carcinoma, Neoplasm Metastasis, Scalp

The skin is one of the largest organs of the human body; however, cutaneous metastases are occasional.^1^ The overall incidence of cutaneous metastasis due to malignancies is 5.3%.^1,2^ The most common cutaneous metastases are associated with breast cancer, followed by lung, colorectal, renal, ovarian, and bladder malignancies.^2,3^

Gall bladder carcinoma (GBC) accounts for 80-95% of biliary tract malignancies and carries a poor prognosis.^2,3^ Most patients remain asymptomatic until the tumor reaches an advanced stage or is incidentally diagnosed. Primary gallbladder carcinoma spread by direct extension and metastasis. By direct extension, the liver is the most usually affected organ, with an incidence of 60 to 90%, while regional lymph nodes are involved in approximately 60% of cases. Extra-abdominal metastasis is rare and is disseminated through vascular dispersion and tumor cell homing. Cutaneous metastasis of primary gallbladder cancer is sporadic and has an incidence of 0.7 to 0.9%.^4^

GBC prevalence varies geographically and racially. The indigenous Mapuche people of Chile have the greatest incidence, with 27.3 per 100,000 person-years documented among females. High rates have also been discovered in regions of India, eastern Asia, and various central and eastern Europe countries. GBC is more common in females with gallstones in Chile and India. It is virtually equally common in men in Eastern Asia, although the link to gallstones is significantly weaker, implying regional etiological differences.^2-4^ Gallbladder carcinomas diagnosed at an advanced age carry a poor prognosis.^1,2^ The bile ducts, stomach, duodenum, colon, omentum, abdominal wall, pancreas, and portal vein are involved by direct extension.^2^ Extra abdominal metastases, vascular dissemination, and tumor cell homing are rare. The lung is the most common extra-abdominal site of metastases. Other rare sites of metastases include the heart, orbit, central nervous system, skin, and bone.^3,4^

Nevertheless, metastases to the scalp have not been fully clarified. According to Paget's “soil-seed” hypothesis, the scalp provided a favorable environment for colonizing and surviving gallbladder cancer cells. In addition, the interaction between tumor cells and certain factors secreted from the dermis or epidermis might be extensively involved in the skin (including the scalp) homing mechanism of metastatic cells.^2,3^

The five-year survival rate among patients diagnosed with gallbladder cancer is <5% and the mean survival time in cases of gallbladder cancer with skin metastasis is 7.5 months. The most common histological type of gallbladder cancer with skin metastasis is adenocarcinoma.^5^ Typically, the metastatic lesions are non-tender, erythematous, nodular, or cutaneous or subcutaneous. Our literature search in PubMed showed only a few cases of cutaneous metastasis of gallbladder carcinoma to date, of which only six were reported on the scalp. Skin metastases usually present as nodular lesion invading the dermis and subcutaneous fat, and is less than 3 cm in diameter and most often asymptomatic.^4,5^

Non-pruritic, indurated lesions in patients should always raise suspicion for cutaneous metastases from internal malignancy and undergo biopsy. Rarely, these lesions may be the initial presentation of malignancy. Cutaneous metastases from gallbladder cancer represent an advanced disease associated with an abysmal prognosis.^2,3^ Cutaneous metastasis needs a histopathologic examination, which immunohistochemistry should be accomplished in case of discrepancies for the presumed primary cancer. Fine needle aspiration cytology is also a minimally invasive diagnostic tool, but complete excision is still the method of choice to ensure the best quality of histopathologic examination.

**Figure 1:**
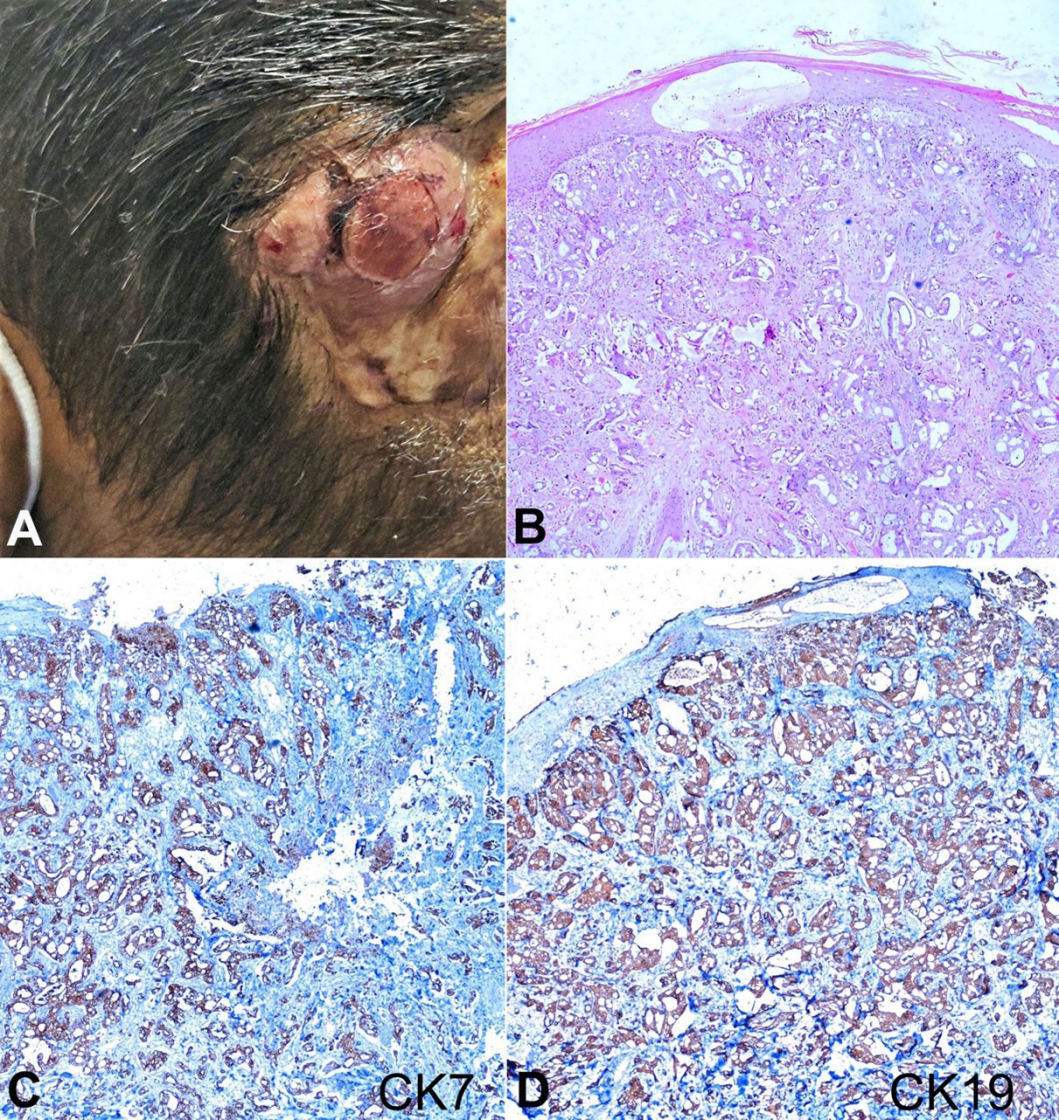
**A –** Gross view of the swelling over the scalp region, with focal ulceration; **B –** Photomicrograph of the nodule biopsy shows thinned-out keratinized stratified squamous epithelium. Sub-epithelium shows infiltration by the adenocarcinoma with an intermixed area of fibrosis and mild chronic inflammation. The tumor is reaching up to the overlying skin (H&E, 100X); **C –** The tumor cells are immunopositive for cytokeratin 7 (CK-7) (100X); **D –** Immunohistochemistry for CK-19 is positive in tumor cells (200X).

Figure 1 refers to a 68-year-old female who presented to the surgery outpatient department (OPD) with swelling over the scalp (Figure 1A) over the last two months. No contributory history was identified. Scalp swelling was fixed, firm, and hard in the right parietal region. No involvement of the underlying bone was observed. On systemic examination, the respiratory system, cardiovascular system, and abdominal examination were within normal limits. Alpha-fetoprotein and liver function tests were within normal limits. Blood investigations revealed microcytic hypochromic anemia and neutrophilic leukocytosis. ESR was two times the reference range. In view of the clinical suspicion of malignancy, a biopsy was taken. Histologic sections showed a skin-covered tissue with focal ulceration and a tumor that extended to the dermis and subcutis (Figure 1B). Tumor cells were arranged in glandular patterns and islands. Individual tumor cells were oval to cuboidal, with a large nucleus, irregular nuclear membrane, vesicular chromatin, prominent nucleoli, and moderate cytoplasm. A few cells showed cytoplasmic clearing. The peritumoral desmoplastic reaction was also identified with intratumorally brisk mitotic figures. Focal areas of necrosis with acute and chronic inflammatory infiltrate were also identified. Immunohistochemistry revealed tumor cells to be diffusely positive for cytokeratin 7 (CK7) and CK19 (Figure 1C and 1D) and negative for CK20, thyroid transcription factor (TTF-1), Gross Cystic Disease Fluid Protein-15 (GCDFP-15), mammaglobin, Hepatocyte Paraffin 1 (Hep Par1), Paired box gene 8 (PAX-8) and Wilms tumor gene 1 (WT1).

An abdominal computed tomography scan on imaging workup revealed a gallbladder mass with multiple liver lesions and brain metastasis. Based on immunohistochemistry and imaging findings, the diagnosis was metastatic gall bladder carcinoma to the scalp.
